# Dissecting the mechanisms of MASLD fibrosis in the era of single-cell and spatial omics

**DOI:** 10.1172/JCI186421

**Published:** 2025-09-16

**Authors:** Fabio Colella, Neil C. Henderson, Prakash Ramachandran

**Affiliations:** 1 Institute for Regeneration and Repair, and; 2 MRC Human Genetics Unit, Institute of Genetics and Cancer, University of Edinburgh, Edinburgh, United Kingdom.

## Abstract

Metabolic dysfunction–associated steatotic liver disease (MASLD), now the most common cause of chronic liver disease, is estimated to affect around 30% of the global population. In MASLD, chronic liver injury can result in scarring or fibrosis, with the degree of fibrosis being the best-known predictor of adverse clinical outcomes. Hence, there is huge interest in developing new therapies to inhibit or reverse fibrosis in MASLD. However, this has been challenging to achieve, as the biology of fibrosis and candidate antifibrotic therapeutic targets have remained poorly described in patient samples. In recent years, the advent of single-cell and spatial omics approaches that can be applied to human samples have started to transform our understanding of fibrosis biology in MASLD. In this Review, we describe these technological advances and discuss the new insights such studies have provided, focusing on the role of epithelial cell plasticity, mesenchymal cell activation, scar-associated macrophage accumulation, and inflammatory cell stimulation as regulators of liver fibrosis. We also consider how omics techniques can enhance our understanding of evolving concepts in the field, such as hot versus cold fibrosis and the mechanisms of liver fibrosis regression. Finally, we touch on future developments and how they are likely to inform a more mechanistic understanding about how fibrosis might differ between patients and how this could influence optimal therapeutic approaches.

## Liver fibrosis drives adverse clinical outcomes in MASLD

Metabolic dysfunction–associated steatotic liver disease (MASLD) and metabolic dysfunction–associated steatohepatitis (MASH) have become the most common chronic liver diseases (CLDs) worldwide (1, 2). Despite only a minority of patients developing cirrhosis (3), MASH remains the fastest-growing indication for liver transplantation (4) and the leading driver for development of hepatocellular carcinoma (HCC) in the Western world (5). Like other causes of CLD, the iterative liver injury observed in patients with MASLD results in persistent activation of hepatic wound-healing responses, ultimately leading to excessive extracellular matrix (ECM) deposition and resultant hepatic scarring, termed fibrosis. Several studies have shown that degree of fibrosis is the best predictor of adverse clinical outcomes in patients with MASLD (6–9). Regression of fibrosis following therapy led to improved clinical outcomes in a subgroup of patients with MASH (10). This close association has led to improvements in fibrosis becoming a cornerstone of the surrogate efficacy endpoints in interventional clinical trials. However, despite recent encouraging phase III trial data on resmetirom and semaglutide, only a minority of patients (26% and 37%, respectively) showed improvements in fibrosis (11, 12). Hence, more work is needed to achieve adequate antifibrotic efficacy.

A major challenge in identifying antifibrotic therapeutic targets in MASLD is the complexity of human liver fibrosis, which often develops over years or decades and involves multiple pathophysiological processes and cell types. Modern single-cell or single-nucleus RNA sequencing technologies (scRNA-seq and snRNA-seq, respectively) have provided a powerful new lens to examine human liver fibrosis at previously unparalleled resolution. Furthermore, state-of-the-art spatial omics approaches promise to herald the next wave of insights in human MASLD pathogenesis. Here, we review how these approaches have advanced understanding of the mechanisms of fibrosis in MASLD and how they will continue to inform antifibrotic therapeutic target identification in the years to come.

## Single-cell and spatial transcriptomic investigation of liver fibrosis

Modern high-throughput single-cell sequencing technologies have been increasingly adopted to investigate complex chronic diseases. While many early human liver single-cell studies used scRNA-seq to provide new insights into disease-associated cell types (13–15), several groups have now adopted snRNA-seq protocols instead, which offer potential advantages and disadvantages (Table 1). Due to the current lack of human studies combining both scRNA-seq and snRNA-seq, existing datasets likely underrepresent key cellular drivers of disease and the complexity of cellular interactions in MASLD. Of course, single-cell technologies continue to improve apace with evolving methodologies, promising to offer more sensitive, lower-cost gene detection (16). Technological advances have been supplemented by improvements in computational workflows, which now enable most laboratories around the world to perform robust analyses (17). As these newer data generation and analytical methods are applied to human MASLD samples, further biological insights are likely to be garnered.

One challenge with scRNA-seq and snRNA-seq data is the loss of spatial location due to tissue dissociation. In spatially patterned diseases such as fibrosis, an understanding of the spatial context and cell-cell communication within the fibrotic niche is crucial for dissecting the cellular and molecular drivers of disease and identifying novel therapeutic targets. Most human liver single-cell studies have therefore used markers identified from single-cell transcriptomic data in immunohistochemistry or in situ hybridization approaches to map disease-associated cell types into different tissue niches. Despite generating important insights, e.g., in studying human liver zonation patterns (15) or identifying scar-associated cell populations in the fibrotic niche of human cirrhosis (14), such methodologies do not enable the exhaustive comparison of in situ molecular profiles needed to fully dissect the pathological mechanisms driving fibrotic niche expansion and disease progression across the spectrum of MASLD. However, new, more unbiased high-dimensional spatial transcriptomics (ST) methodologies (18, 19) can provide new insights into the pathogenesis of MASLD fibrosis. Sequencing-based approaches directly capture RNA transcripts from tissue while adding spatial barcodes via polyT oligo arrays (Visium) (20), DNA-barcoded beads (Slide-seq and HDST) (21, 22), or barcoded DNA nanoballs (Stereo-seq) (23). Early uses of ST methods in human liver tissue included healthy human liver (24), end-stage cirrhotic livers (25, 26), primary sclerosing cholangiopathy (PSC) (27), and acute liver failure (28), in which the ST profiles of liver metabolic zonation, fibrosis, and regeneration were described. Application of ST to human MASLD is less well established, limited to a small number of samples in patients with low levels of fibrosis (29, 30). Nevertheless, in these studies, the presence of hepatic steatosis appeared to alter the ST profile and zonation patterns (29, 30), suggesting that ST may yield new biological insights when applied to larger patient cohorts.

Notably, current human liver data from capture-based ST methods are largely limited by low spatial resolution, meaning that each spot captures multiple cells, making it difficult to determine which specific cell types are within each spatial domain. In contrast, imaging-based ST approaches measuring 100s to 1000s of individual RNA or protein molecules at cellular or subcellular resolution (31) potentially offer more robust single-cell phenotyping and spatial localization. Such high-plex in situ RNA profiling enabled mapping of proregenerative migratory hepatocytes in acute liver failure (28), disease-associated hepatocytes in fibrotic human liver human liver samples (32), and detailed characterization of immune cell localization in human and murine steatotic liver tissue (29). While MASLD fibrosis has not been studied at scale using in situ RNA profiling, applying these methodologies to archival formalin-fixed, paraffin embedded (FFPE) samples could enable comprehensive single-cell spatial profiling across disease stages. Protein-based spatial approaches (e.g., using high-dimensional antibody staining) offer the opportunity to study 10s to 100s of proteins at single-cell resolution in large patient cohorts, potentially enabling identification of immune cell subpopulations with well-described distinguishing markers. Such approaches have been applied to study MASH, PSC (33), and HCC (34). However, despite continually improving data dimensionality, imaging-based approaches have not yet reached whole transcriptome or proteome coverage, necessitating selection of probe or marker panels based on prior knowledge. Therefore, to uncover fibrosis biology and additional therapeutic targets, these technologies are currently best performed alongside unbiased transcriptomics such as scRNA-seq or snRNA-seq. Moreover, high costs of spatial approaches currently limit wide application, meaning more affordable solutions are needed to truly harness their power for diagnostic and therapeutic applications in liver fibrosis.

## Single-cell approaches unpick cellular drivers of MASLD fibrosis

MASLD and MASH are usually associated with systemic metabolic dysfunction, including obesity, diabetes, hypertension, and dyslipidemia. Excessive energy substrate is associated with de novo lipogenesis in the liver, while dysfunctional adipose tissue results in the release of excessive free fatty acids (35). Eventually, these adaptations overwhelm the liver’s buffering capacity, causing hepatic mitochondrial dysfunction and aberrant adipose tissue–liver crosstalk, leading to accumulation of toxic lipids and reactive oxygen species, and ultimately resulting in mitochondrial ER stress (35–37). This metabolic injury in hepatocytes then triggers cellular activation, death, or senescence, causing stimulation of inflammatory and fibrogenic signalling cascades that propagate activation of nonparenchymal cells (e.g., hepatic stellate cells [HSCs] and macrophages) and ultimately lead to the chronic inflammation and fibrosis characteristic of more advanced disease (38, 39). Hepatocyte lipid metabolism’s role as a central driver of MASLD pathogenesis is emphasized by large-scale GWAS, where the majority of genetic polymorphisms associated with the development and progression of MASLD and liver fibrosis (e.g., *PNPLA3*, *TM6SF2*, *MBOAT7*, and *HSD17B13*) (40) are genes predominantly expressed by hepatocytes in the liver that encode proteins responsible for nutrient processing, lipid handling, and the resultant hepatic mitochondrial redox state (41). Hence, much therapeutic focus in MASLD has been placed on targeting metabolic pathways in hepatocytes, for example inhibiting de novo lipogenesis (e.g., Aramchol), reducing energy availability (e.g., GLP-1 and/or glucagon agonists), or enhancing lipid handling (e.g., thyroid hormone receptor [THR] β analog [resmetirom], FXR agonist [obeticholic acid], PPAR agonist [lanifibrinor]) (35). Unfortunately, many of these interventions remain unproven in human MASH. What has been less clear in the field are the specific mechanisms and signalling pathways by which injured/dying hepatocytes in human liver result in nonparenchymal cell activation at different disease stages and spatial locations in the liver, how they lead to the establishment of fibrosis and contribute to disease progression, and which specific pathogenic mediators or cell subpopulations can be targeted therapeutically. Single-cell and spatial technologies are now yielding new insights into these key unanswered questions.

### Epithelial cell plasticity.

**Figure 1:**
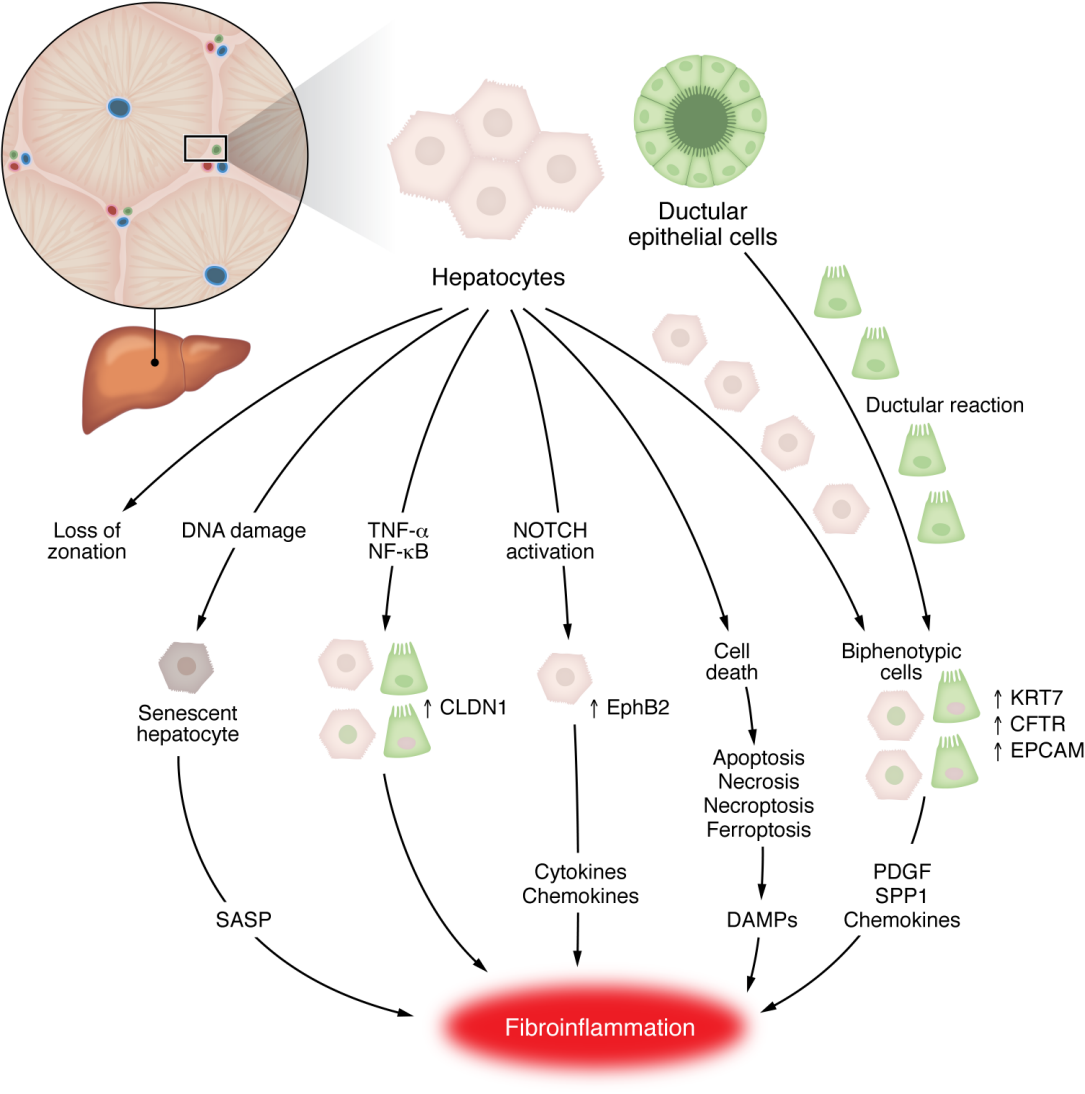
Epithelial plasticity in MASLD. Hepatocytes and ductular epithelial cells (also called cholangiocytes) show extensive transcriptional and phenotypic changes during MASLD pathogenesis. Hepatocytes lose their periportal/pericentral zonation, show an altered expression of tight junction family of proteins, activate NOTCH, become senescent, and undergo cell death. Hepatocytes and ductular epithelial cells give rise to biphenotypic cells that express markers of both cell types and are key players of the ductular reaction associated with liver disease. Overall, these changes contribute to the fibroinflammatory response through the release of DAMPs, SASP factors, cytokines, and chemokines. DAMPs, damage-associated molecular pattern; SASP, senescence-associated secretory phenotype. Created in BioRender; https://BioRender.com/a3v1fv2.

Hepatocyte injury is the key trigger of fibroinflammatory responses in MASH, driving a focus on applying single-cell methodologies to better dissect hepatocyte heterogeneity and transcriptional responses in regulating disease pathogenesis (Figure 1). As discussed in Table 1, the implementation of snRNA-seq has circumvented the difficulties of isolating viable hepatocytes from diseased human tissue (14, 42) and provided more clarity on human hepatocyte heterogeneity and transcriptional responses to disease. The most comprehensive study currently available included snRNA-seq data on approximately 70,000 hepatocytes from 47 patients across the full MASLD/MASH disease spectrum with a range of fibrosis stages (43). Hepatocytes showed most transcriptional changes according to disease severity of any cell type, which was most apparent in patients with advanced MASLD cirrhosis. Notably, markers of hepatocyte zonation such as GLUL and ASS1, which distinguish pericentral and periportal hepatocytes in healthy liver, respectively, are progressively more coexpressed in the same hepatocytes as MASLD progresses, as demonstrated by snRNA-seq and immunofluorescent staining (43). This observation mirrors spatial mass spectrometry data, where zonation patterns of lipids in the liver are lost in more advanced human MASLD (44).

This transcriptional reprogramming of hepatocytes in MASLD also resulted in accumulation of a subpopulation coexpressing hepatocyte and biliary epithelial (cholangiocyte) markers (e.g., KRT7, CFTR, EPCAM) that progressively expanded with MASLD severity and potentially derive from hepatocytes (43). However, significant plasticity was also observed in the cholangiocyte compartment, with expansion of cholangiocytes coexpressing hepatocyte markers (e.g., ALB, ASGR1, TTR, ASS1, PCK1, ABCC2, GPC5, HNF4α) noted across the MASLD spectrum (43). These biphenotypic cholangiocytes likely represent expanded biliary epithelial cells, key to the “ductular reaction” that has been demonstrated to be functionally important in hepatocellular regeneration following chronic injury in mice (45–48). In human MASLD, the association between ductular reaction and increased fibrosis is well recognized (49–51), while in rodent models of CLD, these biphenotypic ductular cells have been shown to promote myofibroblast activation, ECM deposition, and inflammatory cell infiltration (52–56) via secretion of key mediators such as PDGF (57), osteopontin (58), and chemokines (56, 59–61). Due to their transcriptional similarities and coexpression of both hepatocyte and cholangiocyte markers, it remains unclear whether hepatocyte-derived and cholangiocyte-derived biphenotypic epithelial cells exert functional differences in regulating fibrosis. These populations could feasibly have a distinct spatial location and local cellular niche regulating their functions. Application of high-resolution ST in MASLD tissue samples will hopefully shed further light on this.

Hepatic expression of claudin 1 (CLDN1), a member of the tight junction family of proteins, was increased in patients with MASLD (and other etiologies of CLD) and correlated with more advanced fibrosis (62). scRNA-seq and snRNA-seq data localized CLDN1 expression to hepatocytes, cholangiocytes, and biphenotypic epithelial cells as well as HSCs (62). Notably, inhibition of CLDN1 in a range of in vivo and in vitro models abrogated fibrosis and HCC formation, potentially due to reduced cellular plasticity, inhibition of ductular reaction, as well as more direct effects on myofibroblast activation and ECM production (62). Inhibition of CLDN1 via monoclonal antibody was noted to be safe in nonhuman primates (62), with an active phase II clinical trial evaluating CLDN1 inhibition in patients with head and neck cancer (Clinicaltrials.gov NCT06054477). Hence, CLDN1 inhibition is potentially an appealing target for modulation of fibrosis in MASLD.

Notch signalling was shown to increase in hepatocytes from patients with MASH and fibrosis, while in a longitudinal analysis, patients who responded to the treatment in the PIVENS trial (pioglitazone versus vitamin E versus placebo) (63) demonstrated reduced hepatocyte Notch activation (64). In mouse models of MASLD, inhibition of hepatocyte Notch reduced fibrosis despite no change in hepatocyte injury or steatosis, while overexpression of Notch exacerbated fibrosis (64). snRNA-seq on human and mouse MASLD liver tissue identified expansion of a MASH-associated hepatocyte subpopulation expressing high levels of the activation receptor tyrosine kinase ephrin type B receptor 2 (EphB2) (65). EphB2 was shown to be a downstream transcriptional target of the Notch pathway and promoted inflammatory cytokine and chemokine secretion from hepatocytes; accordingly, inhibition of hepatocyte EphB2 expression in a mouse MASH model reduced inflammatory cell recruitment and attenuated fibrosis (65). Hence, pathological Notch signalling may connect hepatocyte injury, inflammation, and fibrosis in MASH.

Beyond transcriptional changes, lipotoxicity in MASLD can drive hepatocyte death, which regulates local inflammatory and fibrogenic responses (66). Specifically, hepatocyte apoptosis was associated with more advanced MASH and fibrosis (67) and suggested to promote disease progression (68). Caspase inhibitors, which inhibit apoptosis and attenuate liver fibrosis in rodent MASH models (69, 70), were tested in clinical trials for MASLD, albeit with disappointing results so far (71). Alternative forms of programmed cell death may also be relevant; for example, necroptosis has been suggested as a predominant driver of cell death in MASLD (66). Interestingly, necroptotic (but not apoptotic) hepatocytes in MASH livers upregulate the “don’t eat me” molecule CD47, while hepatic macrophages show increased expression of the CD47 ligand SIRPα (72). Inhibiting either CD47 or SIRPα improved necroptotic hepatocyte clearance and attenuated fibrosis, highlighting this axis as a possible therapeutic target (72). Dead or dying hepatocytes may also signal directly to HSCs to promote a profibrogenic phenotype, e.g., via release of mitochondria-derived damage-associated molecular patterns (DAMPs) (73), secretion of high-mobility group box-1 (HMGB1) (74), or activation of the purinergic receptor P2Y14 on HSCs through the production of UDP-glucose and UDP-galactose (75). Targeting downstream fibroinflammatory responses to hepatocyte death might prove a more specific and tractable antifibrotic therapeutic option than global inhibition of cell death pathways, with lower potential for off-target effects or inducing the persistence of premalignant epithelial cells.

A fraction of hepatocytes develop a senescent phenotype, a state of permanent cell cycle arrest. Hepatocyte senescence, likely induced by DNA damage and telomere shortening, has been shown to correlate with fibrosis stage and predict adverse clinical outcomes in patients with MASLD (76). Senescent hepatocytes secrete a range of autocrine and paracrine factors (called the senescence-associated secretory phenotype [SASP]) that can regulate responses of adjacent epithelial and nonparenchymal cells and control local inflammation and fibrosis (77). However, before such concepts can be effectively translated, further data are needed to define the transcriptome, spatial niche, and cellular interaction partners of senescent hepatocytes in human MASH, to dissect the pathological versus protective aspects of this process. High-resolution spatial approaches will likely address these questions.

### Mesenchymal cell activation.

As with other fibrotic disorders, myofibroblasts expand in MASLD liver tissue and adopt ECM-producing, migratory, immunomodulatory, and contractile properties that orchestrate disease progression (78). HSCs become activated following hepatic injury (79) and have been shown to be the main source of myofibroblasts in different mouse models of liver fibrosis, including MASLD (80). Indeed, scRNA-seq analysis from patients with cirrhosis of different etiologies identified a population of PDGFRA^+^ ECM-expressing mesenchymal cells populating the fibrotic niche and predicted to derive from HSCs based on RNA velocity analysis (14). However, transcriptionally distinct populations of vascular smooth muscle cells and portal fibroblasts demonstrated in scRNA-seq studies (14, 81) highlight substantial heterogeneity in the hepatic mesenchymal compartment. HSCs themselves are heterogeneous, with clear patterns of zonation observed across the liver lobule (81, 82). Human HSCs in fibrotic liver can be partitioned into myofibroblastic HSCs (myHSCs), enriched in ECM-related molecules, and cytokine- and growth factor–enriched HSCs (cyHSCs), which express high levels of factors such as HGF (83). In advanced liver disease, cyHSCs, which normally exert protective functions, differentiate into myHSCs to promote disease progression, increased liver stiffness, and the development of HCC (83). The concept of myofibroblast heterogeneity and early activated HSCs/myofibroblasts being as a hub of cytokine and growth factor production before transitioning into a more ECM-producing myofibroblast subpopulation was also identified in rodent MASH- and CCl_4_-induced fibrosis (82, 84). However, while the balance of cyHSCs and myHSCs may influence MASLD pathogenesis, the specific signals regulating this transition between cyHSC and myHSC (and potentially back again) need further study.

Nonetheless, abundant data exist describing the mediators that promote transdifferentiation of quiescent HSCs into ECM-producing myofibroblasts, with TGF-β signalling being the key driver (79, 85). However, off-target effects complicate therapeutic targeting of ubiquitous pathways such as TGF-β. Single-cell approaches and modeling of cell-cell communication potentially enable identification more specific molecules and pathways regulating ECM-producing myofibroblasts (86, 87), for example, PDGF/PDGFRA, TNFSF12/TNFRSF12A, IL-1β/IL-1R1, and AREG/EGFR between scar-associated macrophages (SAMacs) and myofibroblasts or Notch signalling between scar-associated endothelial cells and myofibroblasts in advanced cirrhosis (14). An snRNA-seq study of 9 MASH patients demonstrated a MASH-associated HSC phenotype enriched for autocrine signalling (88). These findings were recapitulated in a mouse MASH model that identified the neurotrophin-3–neuronal receptor tyrosine kinase (NTF3/NTRK3) ligand-receptor pair as an autocrine pathway that promotes fibrogenic activity in HSCs and can be therapeutically inhibited in vivo using LOXO-195, a highly specific NTRK3 kinase domain inhibitor (88). Further recent snRNA-seq and single-cell ATAC-seq data identified transcriptional regulators of HSC activation in MASH, highlighting HSC *SERPINE1* as a cell-autonomous driver of fibrogenic activity (89). Bulk profiling has also informed the identification of novel molecules that promote HSC activation; for example, proteomics revealed elevated soluble folate receptor γ (FOLR3) as a driver of HSC activation in MASH, via modulation of TGF-β signalling (90). The cellular source of FOLR3 in the MASH liver remains uncertain but should become clear in more detailed analyses of scRNA-seq and ST data from human samples. In addition to activating signals, HSCs also demonstrate loss of quiescence signals in MASH. scRNA-seq and ATAC-seq analyses in murine MASH identified NR1H4/FXR activity as a key feature of quiescent HSCs that is lost during activation (91). FXR agonists such as obeticholic acid are being actively tested in patients with MASLD (92) and may provide a therapeutic approach for maintaining HSC quiescence.

snRNA-seq analysis has also identified a senescent HSC subpopulation (93). These senescent HSCs expanded in MASH livers and demonstrated an inflammatory and fibrogenic gene expression profile in both human disease and mouse models (93). Senescent HSCs appeared to derive from activated HSCs and upregulated a series of markers, including urokinase plasminogen activator receptor (uPAR), MRC1/CD206, SLC9A9, PTPRB, and STAB2 (93). Notably, targeting senescent cells using chimeric antigen receptor (CAR) T cells directed at uPAR was shown to attenuate fibrosis in a mouse MASH model (94). However, uPAR expression is not specific to senescent HSCs, so it remains uncertain whether selective targeting of senescent HSCs will attenuate or potentially exacerbate fibrosis by promoting the persistence of ECM-producing myofibroblasts (95).

A subpopulation of portal fibroblasts with mesenchymal stem cell features (PMSCs) was identified in mice using scRNA-seq (96). PMSCs and PMSC-derived myofibroblasts expressed a gene signature (*Col1a2*, *Col15a1*, *Igfbp6*, *Loxl1*, *Mgp*, *Thy1*, *Slit2*) that facilitated distinction from HSCs. *Slit2* in particular was specific to PMSCs, and SLIT2^+^ myofibroblasts were identified in the fibrotic niche of cirrhotic human liver of varying etiologies including MASLD, suggesting that PMSC-derived myofibroblasts may contribute to scar deposition in human MASLD (96). Spatially, SLIT2^+^ PMSC-derived myofibroblasts were found adjacent to vessels and in close proximity to SLIT2^–^ myofibroblasts (presumed to be HSC derived) in fibrotic human liver, while SLIT2 itself has been shown to promote HSC activation (96, 97). This suggests that interactions between different mesenchymal cell types may regulate fibrogenesis in CLD. The precise role of this phenomenon in human MASLD pathogenesis remains to be determined.

### Chronic inflammation and SAMac accumulation.

**Figure 2:**
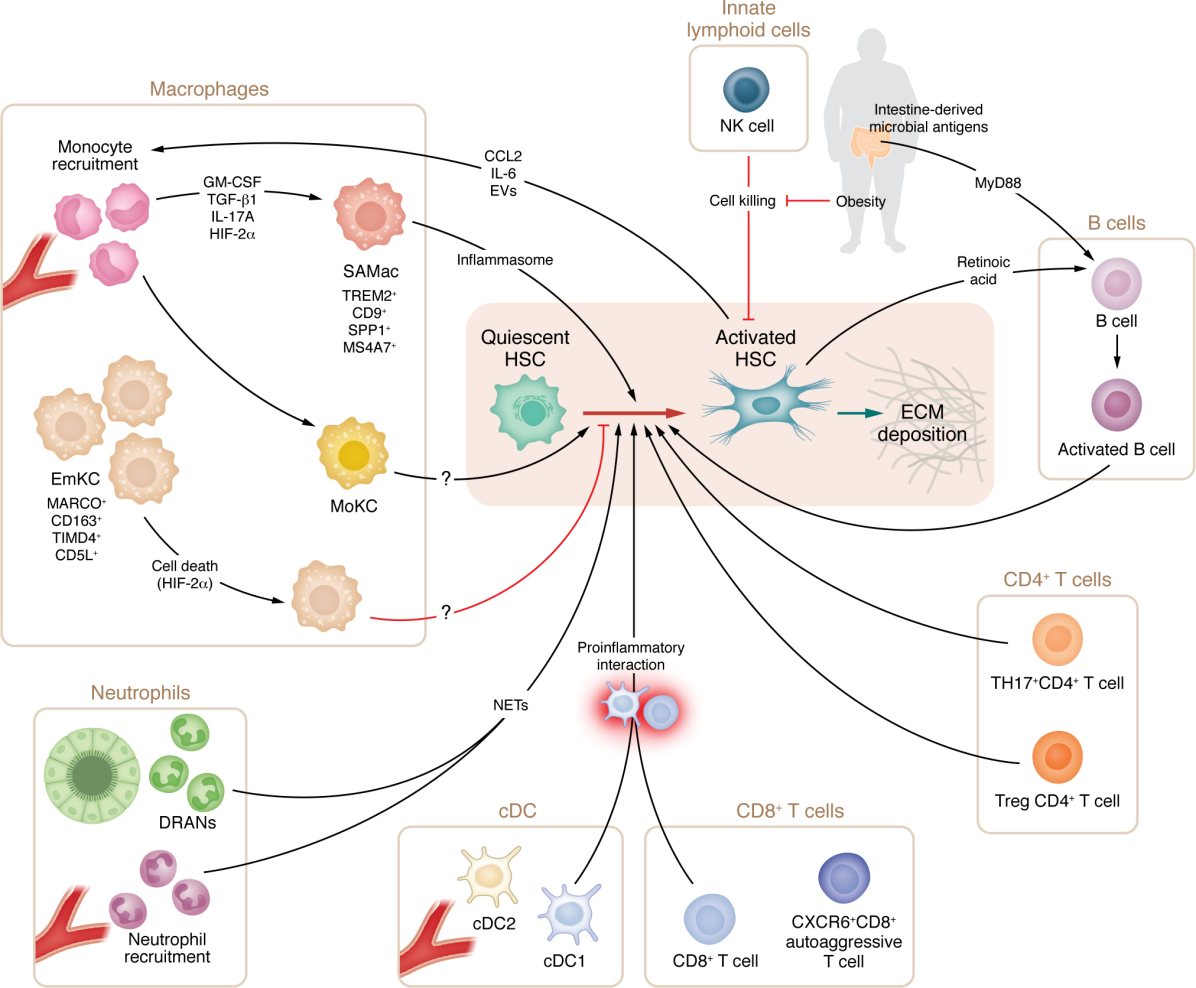
The role of immune cells in MASLD fibrosis. The activation of HSCs, which are responsible for ECM remodeling and fibrosis progression, is tightly controlled by myeloid and lymphoid cells. After injury, monocytes migrate to the liver, where they differentiate into SAMacs, which promote HSC collagen deposition. HSCs can promote additional monocyte recruitment through the secretion of CCL2, IL-6, and extracellular vesicles. DRANs and recruited neutrophils modulate monocyte and HSC activation through NET production. T cells also contribute to tissue injury and HSC activation through a proinflammatory interaction between CD8^+^ T cells and cDC1s as well as through the release of IL-17 and AREG from TH17^+^ and CD4^+^ Treg cells, respectively. B cells become activated by intestine-derived microbial antigens and HSC-secreted retinoic acids, acquiring a proinflammatory phenotype. NK cells can kill activated HSCs and thus promote fibrosis regression, a role that has been shown to be inhibited by obesity. SAMac, scar-associated macrophage; emKC, embryologically derived Kupffer cells; moKC, monocyte-derived Kupffer cells; DRANs, ductular reaction–associated neutrophils; NETs, neutrophil extracellular traps; cDC, classical dendritic cell; Treg, regulatory T cell; HSC, hepatic stellate cell; ECM, extracellular matrix. Created in BioRender; https://BioRender.com/u1u409p.

Chronic inflammation is a key feature of MASLD and its fibrotic microenvironment (Figure 2). The innate immune system has been a major focus of scRNA-seq studies, particularly cells of the monocyte-macrophage lineage that strongly regulate fibrosis in preclinical models (98–101). Initial studies identified a distinct population of TREM2^+^CD9^+^SPP1^+^GPNMB^+^ macrophages that expand in cirrhotic liver and accumulate in the fibrotic niche (14). These SAMacs are derived from the recruitment and differentiation of monocytes rather than resident liver macrophages (Kupffer cells, KCs) and have been shown to promote HSC activation and proliferation in vitro (14, 102), suggesting a potential target population for antiinflammatory and antifibrotic therapies. Notably, transcriptionally similar SAMac populations were also described in fibrosis in other organs, suggesting conserved pathophysiological mechanisms between different fibrotic diseases (103). To confirm that accumulation of SAMacs in the fibrotic niche is not simply a feature of end-stage cirrhosis, deconvolution of bulk liver RNA-seq data across the full MASLD disease spectrum using annotated reference scRNA-seq data demonstrated that SAMac expansion correlates with fibrosis in earlier-stage disease (14), and that accumulation of SAMacs was associated with adverse clinical outcomes in patients with MASLD (8). Additionally, circulating levels of TREM2, a characteristic SAMac marker, shows promise as a serum biomarker of fibrosis in MASLD (104). Overall, these data highlight the potential role of SAMacs in the evolution of fibrosis in MASLD and other causes of CLD.

A population of TREM2^+^CD9^+^SPP1^+^GPNMB^+^ macrophages known as lipid-associated macrophages (LAMs) that are transcriptionally similar to SAMacs was also reported in various mouse models of MASLD (102, 104–107). Spatial analysis using high-plex in situ hybridization, antibody staining, and unbiased ST localized LAMs adjacent to bile ducts in healthy liver and in areas of steatosis in the MASLD liver (29), suggesting that monocytes recruited into areas of tissue injury may differentiate into LAMs/SAMacs within this niche.

To interrogate the mechanisms by which SAMacs regulate fibrosis, ligand-receptor interaction analyses from scRNA-seq data have been used to dissect candidate ligands expressed by SAMacs that are predicted to signal to HSCs/myofibroblasts to promote activation and/or proliferation (86, 87). A combination of soluble mediators, including GM-CSF, IL-17A, and TGF-β1 induced SAMac differentiation from circulating monocytes in vitro, while in vivo blockade of these mediators in the mouse carbon tetrachloride (CCl_4_) CLD model attenuated SAMac differentiation (102). The effect was most striking for TGF-β1 inhibition, where HSC activation and SAMac number were reduced in models of CLD and lung injury (102), indicating that both are at least partially dependent on TGF-β signalling.

Spatially resolved high-plex immunostaining of human biopsies identified a IBA1^+^CD16^lo^CD163^lo^ subpopulation of disease-associated macrophages derived from monocytes and spatially located in portal areas in close proximity to the KRT19^+^ ductular cells in patients with advanced MASH fibrosis, as well as other causes of CLD, including PSC (33). This close spatial relationship suggests that these cells could have functional relevance in the ductular reaction, given that macrophages are known to regulate the ductular reaction in mice (108) via secretion of soluble mediators such as Wnts (109) or TWEAK (110). Furthermore, macrophage-hepatocyte crosstalk can directly control hepatocyte mitochondrial function, lipid accumulation (111), and clearance of senescent hepatocytes (112), all important factors in epithelial dysfunction observed in MASLD (see above). How these direct epithelial-macrophage interactions can be modulated to abrogate fibrosis should be a focus of future work.

Macrophages are producers of inflammatory mediators, including activation of the NLRP3 inflammasome (resulting in release of proinflammatory cytokines IL-1β and IL-18), an important driver of fibrosis in MASLD models (113, 114). The transmembrane molecule membrane-spanning 4-domains A7 (MS4A7) was identified in TREM2^+^ SAMacs from MASLD livers, and MS4A7 deletion in mouse MASH reduced SAMac expansion, liver inflammation, HSC activation, and fibrosis (115). Lipid droplets derived from steatotic hepatocytes were shown to promote SAMac differentiation, MS4A7 expression, and NLRP3 inflammasome activation, with inflammasome activation being at least partially dependent on MS4A7 expression in a cell-intrinsic manner (115). These data potentially provide a mechanistic link between hepatocellular injury, SAMac differentiation, inflammation, and fibrosis; MS4A7 therefore warrants further exploration as a therapeutic target in human MASLD. The transcription factor EGR2 (116) and Notch signalling (117) were both also recently implicated in SAMac differentiation and fibrogenesis in MASLD mouse models. However, current studies aimed at investigating molecular drivers of liver fibrosis are mainly based on mouse models and underestimate the complexity of interactions regulating fibroinflammatory processes in MASLD. Application of spatial omics technologies in human MASLD samples should help clarify these interactions.

Some molecules expressed by SAMacs appear to have antiinflammatory antifibrotic functions in MASLD. The efferocytosis receptor TREM2 is a prime example, as several groups have shown that TREM2 deficiency exacerbates liver inflammation and fibrosis in MASLD models (118, 119), suggesting that TREM2 agonism may be an effective therapeutic strategy. The complexity of TREM2 in the liver is further highlighted by the presence of TREM2^+^ macrophages in healthy human livers, albeit at a lower proportion than in MASH (120), while resident KCs were recently reported to upregulate TREM2 in certain inflammatory contexts (121). Hence, despite numerous candidate antifibrotic targets expressed by SAMacs, it remains unclear which candidates are adequately specific to pathogenic macrophages and selectively inhibit profibrotic functions without disrupting their role in physiological repair and fibrosis regression.

Given their role in lipid metabolism and pathogen clearance, tissue-resident KCs may also have a role in MASLD pathogenesis. In rodent models, embryologically derived KCs (EmKCs) are the main macrophage population in healthy livers but undergo transcriptional reprogramming and cell death in the context of MASLD (122, 123). scRNA-seq has identified two major subsets of EmKCs: CD206^lo^ESAM^–^ KC1, characterized by the expression of immune signatures, and CD206^hi^ESAM^+^ KC2, which are involved in metabolism (124). Notably, KC2 ablation or depletion of the fatty acid transporter CD36 in this subset prevented diet-induced obesity (124). The presence of similar KC subpopulations in human MASLD is yet to be confirmed. In MASLD, the EmKC niche is repopulated with monocyte-derived macrophages that acquire a KC-like phenotype, termed MoKCs (106, 122). Interestingly, there are some suggestions that MoKCs remain functionally distinct from EmKCs, with a more pronounced inflammatory profile and increased liver injury (122, 125). The transcription factor HIF-2α was shown to simultaneously promote KC death and inflammatory activation of monocyte-derived macrophages in MASH, while deletion of HIF-2α protected against inflammation and fibrosis both in vivo and in vitro (126). Whether it is feasible to rebalance the aberrant macrophage compartment in human MASH remains unknown but should be the focus of future studies.

Of course, the chronic inflammatory microenvironment in MASH livers includes numerous other innate and adaptive immune cell types, which have also been studied using single-cell approaches and have variously been associated with the propagation of fibrosis (summarized in Table 2 and Figure 2). More detailed evaluation of which cell populations are the most pertinent drivers of fibrosis at different stages of human MASLD will help rationalize which aspects of this complex inflammatory milieu represent tractable antifibrotic therapeutic targets.

### Vascular reprogramming.

CLD pathogenesis is accompanied by vascular remodeling, which can contribute to fibrosis and portal hypertension (127). scRNA-seq in advanced CLD identified CD34^+^ACKR1^+^ and CD34^+^PLVAP^+^ scar-associated endothelial cell subpopulations that could regulate immune cell recruitment and drive HSC activation through PDGF and NOTCH pathways (14). Specifically in MASLD, changes in the liver sinusoidal endothelial cells (LSECs), the main endothelial population lining the hepatic sinusoids, have been reported to promote steatosis, hepatic inflammation, and fibrosis (128–130). Interestingly, in rodent models, LSEC dysfunction appears before established fibrosis (131, 132), while inhibiting LSEC maladaptation via eNOS activators (133) or targeting epigenetic reprogramming (134) attenuates liver fibrogenesis. However, a detailed study defining the molecular changes in human LSEC during different stages of MASLD is still lacking.

## Systemic drivers of MASLD pathogenesis

**Figure 3:**
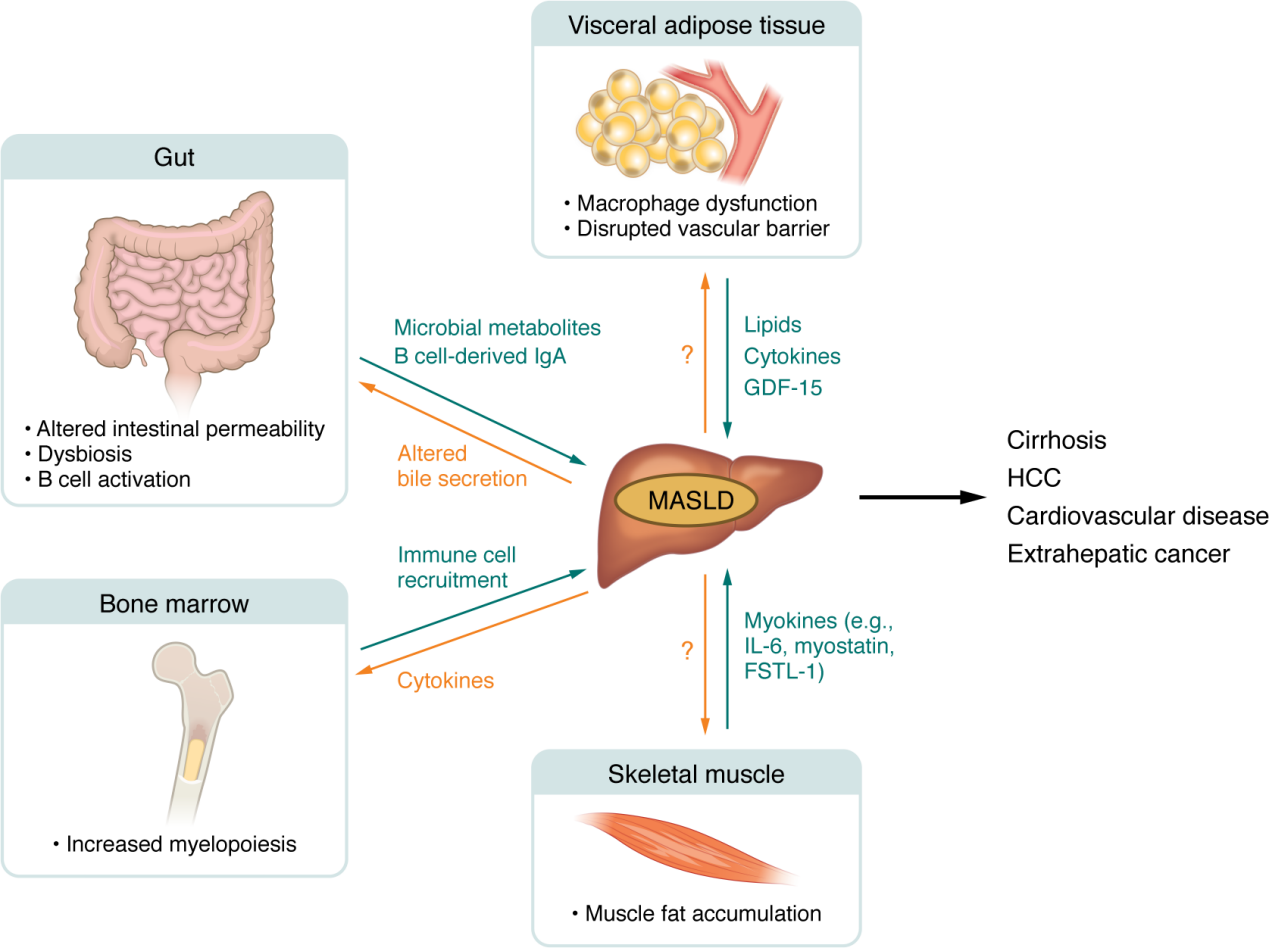
Systemic regulators of MASLD pathogenesis. MASLD is part of a multisystem disorder that occurs concomitantly with liver disease. Changes in the gut, visceral adipose tissue, skeletal muscle, and systemic inflammation drive changes and disease progression in the liver through the release of lipids, cytokines, chemokines, myokines, and microbial metabolites. Ultimately, chronic systemic metabolic dysfunction can lead to intra- and extrahepatic manifestations, including cirrhosis, HCC, cardiovascular disease, and cancer. MASLD, metabolic dysfunction–associated steatotic liver disease; HCC, hepatocellular carcinoma. Created in BioRender; https://BioRender.com/9h9c29q.

MASLD is increasingly recognized as the hepatic component of a systemic disease, with an increased risk of cardiovascular disease and extrahepatic malignancies observed in patients with MASLD (135). However, the cellular and molecular connections between the diseased liver, its manifestations in other tissues, and their reciprocal responses are only starting to be elucidated (136–138) (Figure3). For instance, adipose tissue dysfunction is associated with MASLD development, as demonstrated in lipodystrophic mice in which the redirection of the lipid surplus to the liver led to steatohepatitis (139–141). Interestingly, scRNA-seq analysis of visceral adipose tissue in patients with MASLD showed a change in macrophage phenotype and disruption of vascular barrier integrity, suggesting enhanced systemic release of inflammatory mediators that may signal between adipose tissue and the liver (142). Adipose tissue macrophages were shown to secrete GDF-15 during the early stages of obesity and type 2 diabetes, while hepatocytes upregulated GDF-15 in the liver in during MASH (143). Indeed, GDF-15 has been suggested as a circulating biomarker of disease and fibrosis stage (144). GDF-15 can attenuate the proinflammatory features of macrophages (144, 145) and may therefore represent a mechanism by which tissue damage influences local and systemic inflammation. Obesity also induces changes in other adipose tissue immune cell populations, including NK cells (146, 147), T cells (148), and B cells (149), with additional potential consequences for liver inflammation and fibrosis.

The gut and its microbiome have also gained increased attention in MASLD pathogenesis (150). Fecal microbiota transplantation (FMT) from patients with MASH to germ-free mice fed a high-fat diet led to exacerbation of steatosis (151). However, how changes in the gut and microbiome drive changes in the liver is currently unknown. The main hypothesis is that dysbiosis can alter intestinal permeability, increasing levels of microbially produced metabolites in the portal circulation, which trigger hepatic inflammation (150, 152). While many microbiota signatures have been associated with MASLD and reviewed elsewhere (150), little is known about the spatiotemporal regulation of cellular and molecular pathways governing the transmission of inflammatory signals between the microbiome, gut, and ultimately the liver.

Skeletal muscle secretes myokines that influence distant organs, impacting insulin sensitivity, glucose, and lipid metabolism (153, 154). Accumulation of muscle fat was associated with the presence of MASH (155), while individual myokines such as IL-6 (156), myostatin (157), or follistatin-like protein 1 (FSTL1) (158) are known to regulate liver fibrosis. Application of scRNA-seq and spatial omics to skeletal muscle of patients with MASLD may yield further insights into muscle-specific features or myokines that can be targeted to attenuate liver fibrosis progression.

## Hot versus cold fibrosis: importance of cell circuits

**Figure 4:**
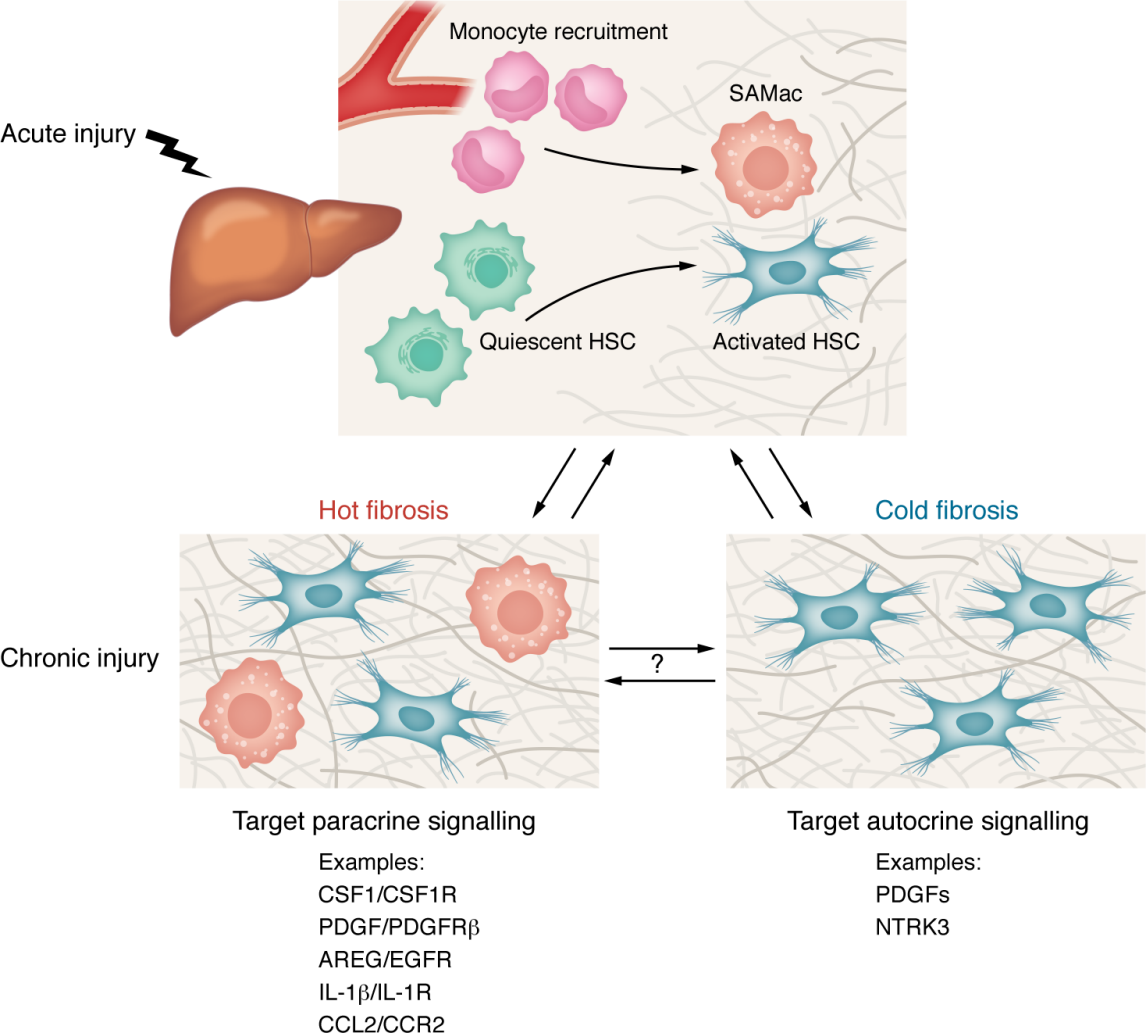
Hot versus cold fibrosis in MASLD. After acute liver injury, recruited monocytes differentiate into SAMacs, which interact with HSCs to orchestrate ECM remodeling and healing. If the injury is prolonged, stable macrophage-fibroblast cell circuit interactions are established that lead to a fibrotic steady state. Two fibrosis states have been proposed: (i) hot fibrosis, characterized by the presence of both cell types and governed by paracrine macrophage-fibroblast interactions, and (ii) cold fibrosis, where only fibroblasts are present and able to self-sustain fibrosis through their autocrine signalling. Future therapeutic approaches could aim to target specific signalling pathways according to the type of fibrosis present. HSC, hepatic stellate cell; SAMac, scar-associated macrophage. Created in BioRender; https://BioRender.com/4pyxtqy.

A key output of scRNA-seq and spatial omics studies is modeling of cellular crosstalk within tissue domains. These analyses have highlighted the importance of macrophage-fibroblast signalling in fibrosis in the liver and other tissues (103). This insight led to the development of a cell circuit model that is predictive of fibrosis progression or healing according to the degree and duration of injury and inflammation (159). Following a short duration of injury, monocyte-derived macrophages accumulate and transiently promote fibroblast activation; if the injury is not sustained, stable macrophage-fibroblast cell circuits are not established and healing occurs. However, if injury is iterative or prolonged, more persistent accumulation of macrophages and fibroblasts then form bistable cell circuits resulting in ECM deposition and establishment of a fibrotic steady state (159). Two distinct fibrotic steady states have been suggested: “hot” fibrosis, characterized by the presence of both macrophages and fibroblasts, and “cold” fibrosis, where only fibroblasts are present. If a patient has hot fibrosis, modulation of inflammation (e.g., by macrophage depletion or by blockade of macrophage-fibroblast signalling) would potentially result in loss of fibroblasts and ECM degradation. In contrast, in the context of cold fibrosis, targeting autocrine fibroblast signalling (e.g., via PDGFs or NTRK3) (160) would be a more effective approach to disrupt disease progression (Figure 4).

Such modeling approaches offer potential conceptual advances in our understanding of fibrosis pathogenesis but are currently largely based on simplified in vitro studies that underrepresent both the number of cell types and complexity of molecular drivers involved. Spatial profiling data have suggested the existence of hot and cold fibrosis in kidney (161) and cardiac disease (162), but whether this paradigm is also relevant for MASLD remains unclear. Going forward, more comprehensive spatial omics analyses of human liver biopsies at different fibrosis stages will be important to determine how hot and cold fibrosis evolve in MASLD. Importantly, better characterization of these spatial and temporal niches, for example by measuring serological ECM components, as was recently proposed (163), could facilitate the identification of circulating biomarkers to decipher the contributions of hot and cold fibrosis and immune-mesenchymal interactions to MASLD pathogenesis in individual patients,

## Fibrosis regression in MASLD – myth or reality?

The potential for fibrosis and even cirrhosis regression has been well described in human liver disease due to chronic viral hepatitis following antiviral therapy (164, 165). In MASLD, detailed phenotyping of patients following bariatric surgery demonstrated that 45.5% of patients with advanced fibrosis (F3–F4) at baseline showed complete resolution after 5 years (166). Importantly, recent data also showed that patients with MASLD exhibiting fibrosis regression following treatment have improved clinical outcomes (10). Hence, fibrosis regression in MASLD should be a realistic goal of therapeutic interventions.

However, the mechanisms orchestrating liver fibrosis regression in human MASLD remain poorly described, with most mechanistic knowledge being derived from rodent models (167, 168). During rodent fibrosis regression, activated HSCs can undergo apoptosis, become senescent, or revert to a quiescent state with downregulation of ECM production (167, 168). Specific subpopulations of monocyte-derived macrophages upregulate matrix-degrading enzymes such as MMP9, MMP12, and MMP13 and antiinflammatory mediators such as TREM2 to abrogate inflammatory activity and enhance fibrosis regression (118, 169–172). These proresolution features of macrophages are promoted by signals, including phagocytosis (169), autophagy (173), nuclear receptor subfamily 4 group A member 1 (NR4A1) activity (174), and TREM2 signalling (118, 121). Interplay with other immune cells can also reprogram macrophages to favor scar resolution. For example, in mouse MASH, neutrophils can promote macrophage reprogramming via the microRNA miR-223, resulting in increased macrophage IL-10 secretion, reduced hepatic inflammation, and accelerated fibrosis regression (175). The inhibition of MAIT cell–macrophage interactions via the administration of acetyl-6-formylpterin enhanced proresolution macrophage accumulation and enhanced fibrosis regression (176), suggesting that MAIT cells may favor a profibrotic macrophage phenotype.

Immune cells may also directly interact with HSCs during fibrosis regression. HSCs express a ligand for NKp46, a major NK cell activating receptor, which enhances HSC apoptosis (177). In addition, NK-driven killing of HSCs via NKG2D and TNF-related apoptosis-inducing ligand (TRAIL) can ameliorate mouse liver fibrosis (178). scRNA-seq identified accumulation of liver CD69^+^CD103^–^CD8^+^ tissue-resident memory (Trm) CD8^+^ T cells during the resolution of murine MASH and associated induction of FasL/Fas-mediated HSC apoptosis with fibrosis regression (179).

However, rodent liver fibrosis models often resolve rapidly, calling into question the translational applicability to human disease. It is therefore imperative to use modern single-cell and spatial approaches to study mechanisms of fibrosis regression in large cohorts of human MASLD liver biopsies.

## Conclusions and future perspectives

As described above, high-resolution omics techniques are transforming our understanding of the mechanisms of fibrosis in MASLD, defining key pathogenic cell types, and identifying candidate therapeutic targets. Crucially, these approaches are being widely applied in human samples, shifting discovery science in MASLD away from imperfect rodent and in vitro models and prioritizing target and biomarker identification in patients. However, omics methods cannot recapitulate “dynamic” aspects of cell-cell interactions in fibrosis, and datasets should continue to be supplemented with functional biology, for example intravital microscopy in rodents or perfusable biochips using human cells. Nevertheless, the advance that single-cell and spatial omics provides is starting to bear dividends, with a range of new therapies being developed and tested in clinical trials.

There remain key unanswered questions to be addressed in the forthcoming years. First, more data during earlier-stage disease is needed to better define pathological cell types and candidate therapeutic targets at fibrosis stages where antifibrotic interventions are more likely to be tractable. Second, factors such as genetics, sex, and ethnicity impact fibrosis heterogeneity and progression. Future omics studies should be conducted in sufficient patient numbers with detailed clinical metadata from different ethnicities and geographical regions, to allow the effects of these host factors on pathophysiological mechanisms to be elucidated. Ideally, such studies will also sample tissue from different body compartments (e.g., adipose tissue, gut, bone marrow), to comprehensively examine the systemic impact of MASLD and its effects on fibrogenesis. Eventually, such studies will potentially enable a more precision medicine–based approach to MASLD, where host factors and the nature of fibrosis (e.g., hot vs. cold) will inform which therapies might be most efficacious in particular individuals. Finally, more detailed cellular and molecular interrogation of fibrosis regression in patient samples will be essential going forward, as ultimately the goal of antifibrotic interventions should be to reverse established disease.

## Figures and Tables

**Table 2:**
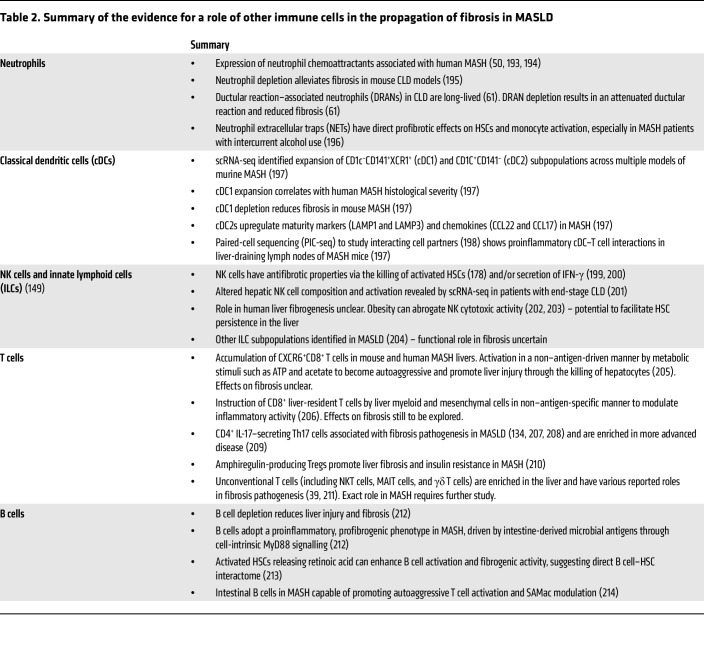
Summary of the evidence for a role of other immune cells in the propagation of fibrosis in MASLD

**Table 1:**
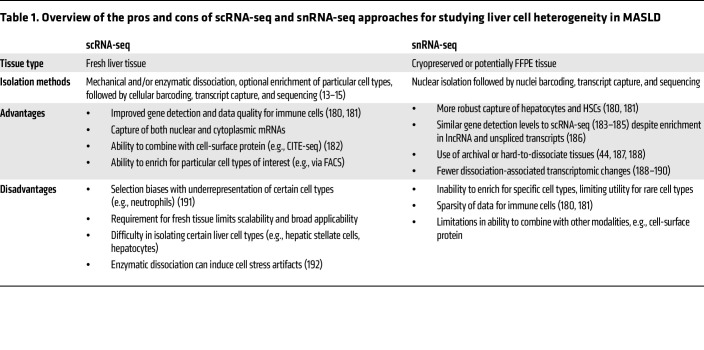
Overview of the pros and cons of scRNA-seq and snRNA-seq approaches for studying liver cell heterogeneity in MASLD

## Version 1 09/16/2025

Electronic publication
